# An Exemestane Derivative, Oxymestane-D1, as a New Multi-Target Steroidal Aromatase Inhibitor for Estrogen Receptor-Positive (ER^+^) Breast Cancer: Effects on Sensitive and Resistant Cell Lines

**DOI:** 10.3390/molecules28020789

**Published:** 2023-01-12

**Authors:** Cristina Amaral, Georgina Correia-da-Silva, Cristina Ferreira Almeida, Maria João Valente, Carla Varela, Elisiário Tavares-da-Silva, Anne Marie Vinggaard, Natércia Teixeira, Fernanda M. F. Roleira

**Affiliations:** 1 UCIBIO, REQUIMTE, Laboratory of Biochemistry, Department of Biological Sciences, Faculty of Pharmacy, University of Porto, Rua Jorge Viterbo Ferreira, n° 228, 4050-313 Porto, Portugal; 2 Associate Laboratory i4HB, Institute for Health and Bioeconomy, Faculty of Pharmacy, University of Porto, 4050-313 Porto, Portugal; 3 National Food Institute, Technical University of Denmark, 2800 Kongens Lyngby, Denmark; 4 Univ Coimbra, CIEPQPF, Faculty of Pharmacy, Laboratory of Pharmaceutical Chemistry, Azinhaga de Santa Comba, Pólo III, Pólo das Ciências da Saúde, 3000-548 Coimbra, Portugal; 5 CIEPQPF, Coimbra Institute for Clinical and Biomedical Research (iCBR), Clinic Academic Center of Coimbra (CACC), Faculty of Medicine, University of Coimbra, Azinhaga de Santa Comba, Pólo III Pólo das Ciências da Saúde, 3000-548 Coimbra, Portugal

**Keywords:** breast cancer, endocrine therapy, endocrine resistance, aromatase inhibitors, exemestane, oxymestane, anti-cancer properties, multi-target compounds, aromatase, estrogen receptor, androgen receptor

## Abstract

Around 70–85% of all breast cancer (BC) cases are estrogen receptor-positive (ER^+^). The third generation of aromatase inhibitors (AIs) is the first-line treatment option for these tumors. Despite their therapeutic success, they induce several side effects and resistance, which limits their efficacy. Thus, it is crucial to search for novel, safe and more effective anti-cancer molecules. Currently, multi-target drugs are emerging, as they present higher efficacy and lower toxicity in comparison to standard options. Considering this, this work aimed to investigate the anti-cancer properties and the multi-target potential of the compound 1α,2α-epoxy-6-methylenandrost-4-ene-3,17-dione (**Oxy**), also designated by Oxymestane-D1, a derivative of Exemestane, which we previously synthesized and demonstrated to be a potent AI. For this purpose, it was studied for its effects on the ER^+^ BC cell line that overexpresses aromatase, MCF-7aro cells, as well as on the AIs-resistant BC cell line, LTEDaro cells. **Oxy** reduces cell viability, impairs DNA synthesis and induces apoptosis in MCF-7aro cells. Moreover, its growth-inhibitory properties are inhibited in the presence of ERα, ERβ and AR antagonists, suggesting a mechanism of action dependent on these receptors. In fact, **Oxy** decreased ERα expression and activation and induced AR overexpression with a pro-death effect. Complementary transactivation assays demonstrated that **Oxy** presents ER antagonist and AR agonist activities. In addition, **Oxy** also decreased the viability and caused apoptosis of LTEDaro cells. Therefore, this work highlights the discovery of a new and promising multi-target drug that, besides acting as an AI, appears to also act as an ERα antagonist and AR agonist. Thus, the multi-target action of **Oxy** may be a therapeutic advantage over the three AIs applied in clinic. Furthermore, this new multi-target compound has the ability to sensitize the AI-resistant BC cells, which represents another advantage over the endocrine therapy used in the clinic, since resistance is a major drawback in the clinic.

## 1. Introduction

In 2020, around 2.3 million new breast cancer cases were diagnosed [1], with about 70–85% of the cases being estrogen receptor-positive (ER^+^). In this type of tumor, estrogens play a pivotal role in cell growth, with endocrine therapy being the main therapeutic approach used in clinic, by blocking estrogen synthesis through aromatase inhibitors (AIs), or preventing the activation of estrogen-signaling pathways, through the use of anti-estrogens [2,3]. In fact, the third-generation of AIs, Anastrozole (Ana), Letrozole (Let) and Exemestane (Exe) are the first-line treatment options in post-menopausal women, with early and metastatic stage, as well as in pre-menopausal women, after ovarian function suppression [4,5,6]. Nevertheless, despite their clinical benefit, they can induce several side effects, and their continued use may lead to the development of acquired resistance, which causes tumor re-growth and disease progression [2]. To improve treatment, some combined therapies with CDK4/6 or mTOR inhibitors and AIs have been applied in clinic. However, despite improving the overall therapeutic outcome, some of these combinations have worrisome cytotoxic effects or do not improve the expansion on overall survival, and as a result resistance continues to occur [2,7,8,9]. Furthermore, it has been reported for CDK4/6 inhibitors that 10% of patients develop de novo resistance, while others develop acquired resistance after 24–28 months when used as first-line therapy or after a shorter period when used as second-line therapy [10,11]. In addition to this concern, it is known that the endocrine therapy with AIs causes loss of bone mineral density, increasing the risk of bone fractures and osteoporosis [12,13]. Clinical application of bisphosphonates with AIs successfully prevents these adverse effects; however, it should be pointed out that their use is associated with some mild-to-severe side-effects [14,15]. Considering all this, the search for new, safer and more effective forms of treatment is crucial to improve therapy for ER^+^ breast cancer.

**Figure 1:**
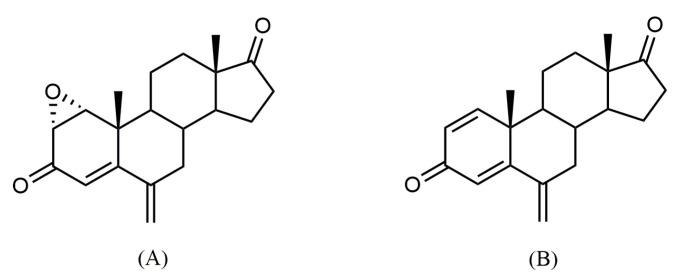
Chemical structures of 1α,2α-epoxy-6-methylenandrost-4-ene-3,17-dione, also known as Oxymestane-D1 (**Oxy**) (**A**) and its parent compound exemestane (Exe) (**B**).

In line with this, our group has designed and synthesized new potent steroidal AIs with promising anti-cancer properties in sensitive ER^+^ breast cancer cells [16,17,18,19,20,21]. Some of these new AIs, in addition to acting on aromatase, have the ability to modulate ERα (selective ER modulator, SERM), as well as the androgen receptor (AR), acting as multi-target drugs in cancer cells [19,21]. Considering the key roles of aromatase and ERs on ER^+^ breast tumors, the anti-cancer drugs with dual AI and SERM properties are pointed as a future therapeutic strategy for ER^+^ breast cancer treatment [22]. In fact, the binding pockets of aromatase and ERs contain equivalent residues, suggesting that they can accommodate the same ligands [3]. In the past, this type of dual therapeutic strategy was studied using the combination of Ana with Tamoxifen, or Let with Fulvestrant, though AIs alone were more effective than when combined with a SERM or a selective ER down-regulator (SERD) [23,24]. Moreover, the uptake of different drugs enhances the risk of drug interactions and consequently leads to more pronounced side effects, which highlights the importance of a single compound with multi-target action [3]. Multi-target drugs are emerging because, in addition to being able to improve the overall tolerance to anti-cancer agents, they are pointed out to be more effective, more potent and less toxic in comparison to the standard options [3,25]. Several groups, including ours, have been working on the search for multi-target drugs for breast cancer treatment [19,21,22,26,27,28,29], with norendoxifen being the first multi-target compound identified [29]. As far as we know, only our group has demonstrated the in vitro anti-cancer properties of some of these molecules in ER^+^ breast cancer cells [19,21,26]. Thus, following this line of research, in this work we investigated the anti-cancer properties and the multi-target potential of the compound 1α,2α-epoxy-6-methylenandrost-4-ene-3,17-dione (Figure 1), also known as Oxymestane-D1 (**Oxy**), which was previously designed and synthesized by our group [20]. **Oxy** was designed with the aim of preparing a molecule that simultaneously inhibits aromatase, but also possesses anti-proliferative activity through other mechanisms. For this, an epoxide function, which is a structural feature associated with anti-proliferative activity [30], was added at position 1,2 of Exe, a very potent AI. Actually, **Oxy** is a potent AI in human placental microsomes (IC_50_ of 0.81 µM) and in ER^+^ breast cancer cells (MCF-7aro) (IC_50_ of 1.18 µM), reducing MCF-7aro cells viability in an aromatase-dependent manner, being in this last case more potent than Exe [20]. In addition, our group demonstrated that **Oxy** can also reduce the viability of lung, liver, colon and prostate cancer cell lines, even being considered to be a more potent anti-cancer molecule than other conventional chemotherapeutic drugs [31].

## 2. Results

### 2.1. Effects of **Oxy** on Proliferation of ER^+^ Breast Cancer Cells

**Figure 2:**
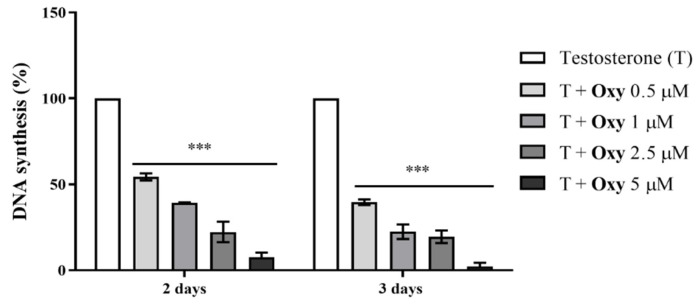
Effects of **Oxy** on proliferation (rate of DNA synthesis) of sensitive breast cancer cells. MCF-7aro cells stimulated with T (1 nM) were treated with different concentrations of **Oxy** (0.5–5 μM), for 2 and 3 days. Cells only stimulated with T were used as control (100% of DNA synthesis). Results are expressed as a mean ± SEM of at least three independent experiments, each performed in triplicate. Significant differences between **Oxy**-treated cells and the control (T-stimulated cells) are shown by *** (*p* < 0.001).

As previously reported by our group [20], **Oxy** significantly reduced the viability of MCF-7aro cells, in a dose- and time-dependent manner. To further understand the anti-cancer properties of the AI **Oxy** on ER^+^ breast cancer cells, its effects on MCF-7aro cell proliferation were explored by performing thymidine incorporation assays, as well as cell cycle analysis. MCF-7aro cells stimulated with T (1 nM) were treated with **Oxy** (0.1–5 µM) for 2 and 3 days. Results demonstrate that this AI dramatically reduced (*p* < 0.001) the rate of DNA synthesis in a dose- and time- dependent manner (Figure 2). Furthermore, as presented in Table 1, this compound impaired the progression of cell cycle by causing a significant (*p* < 0.001) cell cycle arrest on G_0_/G_1_ phase, which consequently significantly (*p* < 0.001) reduced the number of cells on the S phase.

### 2.2. Effects of **Oxy** on Cell Death of ER^+^ Breast Cancer Cells

To understand if the effects of **Oxy** on viability of MCF-7aro cells [20] were a consequence of apoptosis, the translocation of PS to the outer surface of plasma membrane, the activities of caspases-7/-9/-8 and the mitochondrial transmembrane potential (∆Ψm) were investigated in cells treated with **Oxy** at 1 µM for 3 days. This concentration was similar to the IC_50_ value of aromatase inhibition (IC_50_ of 1.18 µM) reported for **Oxy** in this cell model [20].

**Figure 3:**
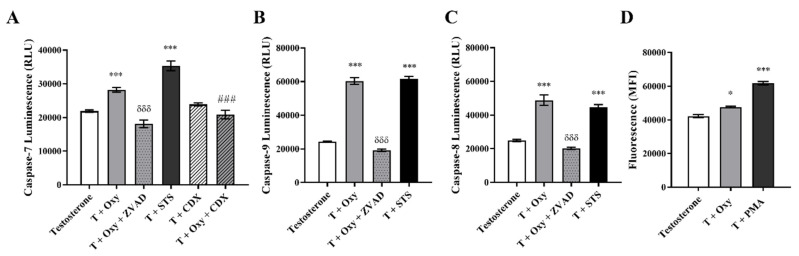
Involvement of apoptosis in the effects induced by **Oxy** on sensitive breast cancer cells. MCF-7aro cells stimulated with T (1 nM) were treated with **Oxy** (1 μM) with or without CDX (1 μM), for 3 days. After treatment, the activities of (**A**) caspase-7, (**B**) caspase-9 and (**C**) caspase-8 as well as (**D**) the intracellular production of ROS were analyzed. Cells treated only with T were considered as a control, while cells treated with STS (10 µM) and PMA (25 ng/mL) were used as positive controls for caspase activation assays and ROS, respectively. Z-VAD-FMK was used as a pan-caspase inhibitor in caspases activation assays. Results are shown as the mean ± SEM of at least three independent experiments, each performed in triplicate. Results are expressed as relative luminescence units (RLU) for caspases activation assays and as mean fluorescence intensity (MFI) for ROS. Significant differences between the control and **Oxy**-treated cells are shown by * (*p* < 0.05) and *** (*p* < 0.001), while differences between **Oxy**-treated cells with or without Z-VADFMK are indicated by *δδδ* (*p* < 0.001) and differences between **Oxy**-treated cells with or without CDX are indicated by ### (*p* < 0.001).

As presented in Figure 3, **Oxy** significantly increased (*p* < 0.001) the activities of caspase-7 (1.29 fold) (Figure 3A), caspase-9 (2.48 fold) (Figure 3B) and caspase-8 (1.96 fold) (Figure 3C) in comparison to control. The activation of all these caspases was significantly (*p* < 0.001) prevented by the incubation with Z-VAD-FMK, a pan-caspase inhibitor used as a negative control. In addition, by analyzing the ∆Ψm, it was observed that **Oxy** induced a 5.66 times (*p* < 0.001) greater ∆Ψm loss, when compared to control (Table 2). As mitochondria dysfunction can be linked with the formation of ROS, its generation was also evaluated. We verified (Figure 3D) that **Oxy** leads to a significant (*p* < 0.05) increase in production of ROS, when compared to control.

Moreover, by labelling MCF-7aro cells with Annexin V, it was detected that **Oxy** caused a significant (*p* < 0.001) increase (3.21 times) in the binding to Annexin V when compared to control (Table 3). This effect was also accompanied by an increase in 7-AAD^+^ cells (*p* < 0.001).

### 2.3. Possible Mechanism of Action of **Oxy**: The Involvement of Aromatase, Estrogen Receptor and Androgen Receptor

Our group had previously described that **Oxy** presents an aromatase inhibition of 88.6% and an IC_50_ value of 1.18 µM in MCF-7aro cells and that it induces an aromatase-dependent reduction in cell viability [20]. Taking this into account and the fact that **Oxy** impaired cell growth and induced apoptosis, we further investigated its targets and mechanism of action by studying the involvement of aromatase, ERs and AR. MCF-7aro cells stimulated with T (1 nM) or E_2_ (1 nM) were treated with **Oxy** (1 µM) with or without the ERα antagonist ICI 182 780 (fulvestrant) (100 nM), ERβ antagonist PHTPP (1 µM) or AR antagonist CDX (1 µM) for 3 days.

**Figure 4:**
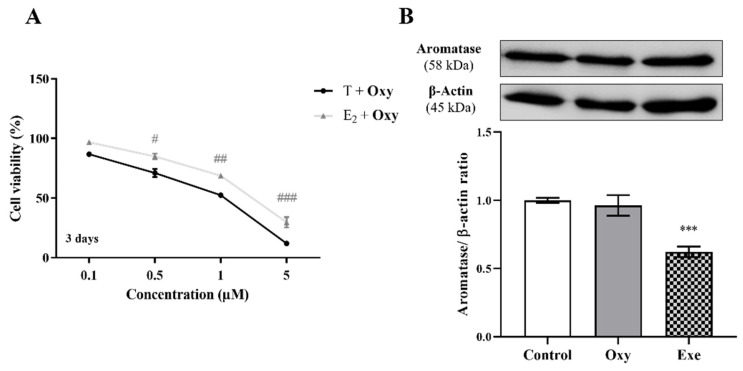
Involvement of aromatase in the effects induced by **Oxy** on breast cancer cells. (**A**) Effects of **Oxy** (0.1–5 μM) on viability of MCF-7aro cells stimulated with T (1 nM) or E_2_ (1 nM), after 3 days of exposure. (**B**) Effects of **Oxy** (1 μM) on aromatase protein expression levels of MCF-7aro cells, after 8 h. Cells without **Oxy** treatment were considered as control. Exe at 10 μM was used as a reference AI. For Western Blot, β-actin was used as a loading control, with the densitometry results presented as aromatase/β-actin ratio. The protein expression obtained for treated cells was normalized in relation to protein expression of control. Results are presented as the mean ± SEM of at least three independent experiments, each performed in triplicate. Significant differences between T-treated and E_2_-treated cells are denoted by # (*p* < 0.05), ## (*p* < 0.01) and ### (*p* < 0.001), while significant differences between the control and Exe-treated cells are shown by *** (*p* < 0.001).

In relation to aromatase, and as previously reported [20], significant differences (*p* < 0.05, *p* < 0.01, and *p* < 0.001 for 0.5, 1 and 5 µM **Oxy**, respectively) were observed between T- versus E_2_-treated cells, after 3 days of treatment (Figure 4A), a behaviour similar to Exe. However, contrary to Exe, **Oxy** caused no significant alteration to the expression levels of aromatase (Figure 4B).

**Figure 5:**
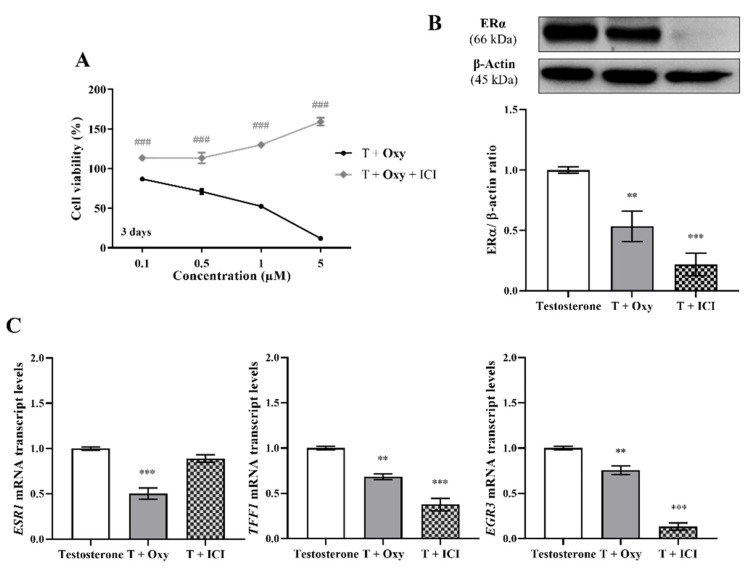
Involvement of ERα in the effects induced by **Oxy** on breast cancer cells. (**A**) Effects of **Oxy** (0.1–5 μM) on viability of MCF-7aro cells stimulated with T (1 nM) and treated with or without ICI (100 nM), after 3 days. Effects of **Oxy** (1 μM) on the expression levels of (**B**) ERα protein or on (**C**) mRNA transcript levels of *ESR1, TFF1, EGR3* and *PDZK1* genes, after 3 days. Cells without **Oxy** treatment were considered as control, while cells treated with ICI (100 nM) were designated as negative control. β-actin was used as a loading control, with the densitometry results presented as ERα/β-actin ratio. The protein expression obtained for treated cells was normalized in relation to protein expression of control (1 nM T). To quantify the mRNA transcript levels of *ESR1, TFF1, EGR3* and *PDZK1* genes, the housekeeping gene *TUBA1A* was used. The mRNA transcript levels of treated cells were normalized in relation to mRNA transcript levels of control (Testosterone). Results are presented as the mean ± SEM of at least three independent experiments, each performed in triplicate. Significant differences between cells treated with **Oxy** with or without ICI are denoted by ### (*p* < 0.001), while between the control and **Oxy**- or ICI-treated cells are shown by ** (*p* < 0.01) and *** (*p* < 0.001).

**Figure 6:**
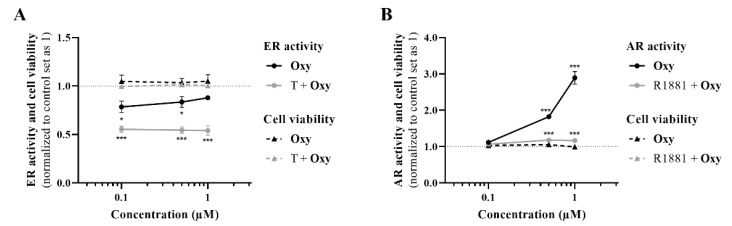
ER and AR transactivation assays. (**A**) Effects of **Oxy** (0.1–1 μM) on ER activation, in the presence or absence of 1 nM T, after 24 h of incubation. (**B**) Effects of **Oxy** (0.1–1 μM) on AR activation, in the presence or absence of 0.1 nM R1881, after 24 h of incubation. Data were normalized to control (cells not treated with **Oxy**), which was set as 1. Results are presented as mean ± SEM of four independent experiments, each performed in triplicate. Significant differences between the control and cells treated with **Oxy** are denoted by * (*p* < 0.05) and *** (*p* < 0.001).

Regarding ERα, the results demonstrated that, in the presence of the ERα antagonist ICI, **Oxy** was unable to reduce cell viability, and a significant increase (*p* < 0.001) in cell viability was even observed when compared with cells only treated with **Oxy** (Figure 5A). Moreover, this compound significantly reduced the protein expression levels of ERα (*p* < 0.01; Figure 5B), as well as the transcript levels of *ESR1* gene (*p* < 0.001; Figure 5C), the gene that encodes ERα, with this latter effect not observed when cells are treated with ICI. In addition, similarly to ICI, the new AI significantly decreased the transcript levels of the ERα-regulated genes (Figure 5C), *TFF1* (*p* < 0.01), *EGR3* (*p* < 0.01) and *PDZK1* (*p* < 0.001). Data on ER transactivation assay showed that **Oxy** acts as a potent ER antagonist in the presence of T (Figure 6A), with over 40% inhibition of T agonistic effect for all concentrations of **Oxy** tested (*p* < 0.001). Some inhibition was observed for the two lowest concentrations in the absence of the ER agonist (*p* < 0.05), meaning that **Oxy** does not appear to detain any ER agonistic potential. Furthermore, no effect on cell viability was observed under any of the tested conditions (Figure 6A).

**Figure 7:**
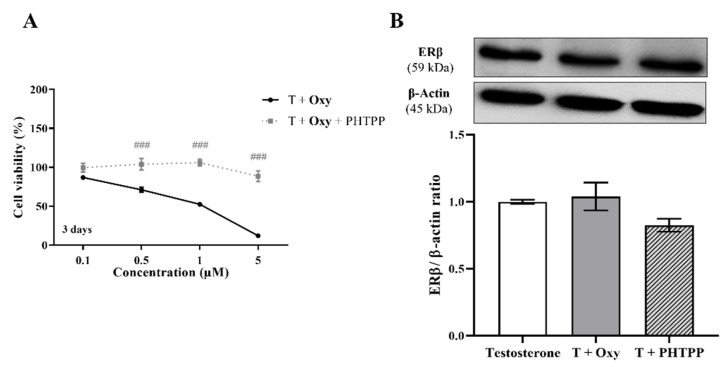
Involvement of ERβ in the effects induced by **Oxy** on breast cancer cells. (**A**) Effects of **Oxy** (0.1–5 μM) on viability of MCF-7aro cells stimulated with T (1 nM) and treated with or without PHTPP (1 µM) after 3 days. (**B**) Effects of **Oxy** (1 μM) on the expression levels of ERβ protein after 3 days. Cells without **Oxy** treatment were considered as control, while cells treated with PHTPP (1 µM) were designated as negative control. β-actin was used as a loading control, with the densitometry results presented as ERβ/β-actin ratio. The protein expression obtained for treated cells was normalized in relation to protein expression of control (1 nM T). Results are the mean ± SEM of at least three independent experiments, each performed in triplicate. Significant differences between cells treated with **Oxy** with or without PHTPP are denoted by ### (*p* < 0.001).

On the other hand, in relation to the involvement of ERβ, our results showed that in the presence of the ERβ antagonist PTHPP, **Oxy** did not affect cell viability when compared to control (Figure 7A). Thus, significant differences (*p* < 0.001) in cell viability between **Oxy**-treated cells with and without PHTPP were noticed. Nevertheless, **Oxy** did not affect the expression levels of ERβ protein (Figure 7B) when compared to control.

**Figure 8:**
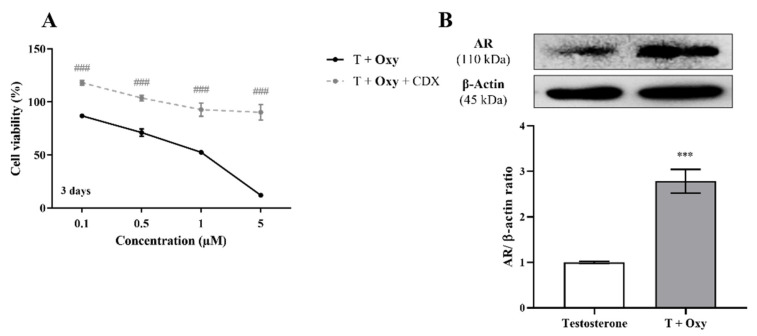
Involvement of AR in the effects induced by **Oxy** on breast cancer cells. (**A**) Effects of **Oxy** (0.1–5 μM) on viability of MCF-7aro cells stimulated with T (1 nM) and treated with or without CDX (1 µM) after 3 days. (**B**) Effects of **Oxy** (1 μM) on the expression levels of AR protein after 3 days. Cells without **Oxy** treatment were considered as control. β-actin was used as a loading control, with the densitometry results presented as AR/β-actin ratio. The protein expression obtained for treated cells was normalized in relation to protein expression of control (1 nM T). Results are the mean ± SEM of at least three independent experiments, each performed in triplicate. Significant differences between cells treated with **Oxy** with or without CDX are denoted by ### (*p* < 0.001), while between the control and **Oxy**-treated cells are shown by *** (*p* < 0.001).

In relation to the involvement of AR on **Oxy** action, results demonstrated that when AR is blocked by CDX, a significant (*p* < 0.001) increase in cell viability is detected when compared with cells treated only with **Oxy** (Figure 8A). In addition, by Western blot, it was demonstrated that this new molecule has the ability to significantly (*p* < 0.001) increase the expression levels of AR when compared to control (Figure 8B). To understand the role of this AR overexpression, the activity of caspase-7 was evaluated when AR was blocked and, as presented in Figure 3A, CDX significantly (*p* < 0.001) prevented the activation of caspase-7 induced by **Oxy**. AR activity of **Oxy** was confirmed through the AR-EcoScreen™ transactivation assay. As presented in Figure 6B, results show that **Oxy** acts as an AR agonist, with an induction of 1.8 and 2.9 over control for 0.5 and 1 µM **Oxy**, respectively (*p* < 0.001). Moreover, an induction of AR was also observed when cells were co-exposed to **Oxy** and the AR agonist R1881 (around 1.2-fold increase over control for the two highest concentrations tested; *p* < 0.001), which means that **Oxy** appears not to detain antagonistic potential. In addition, it should be pointed out that the concentrations studied were not cytotoxic in this cell model (Figure 6B).

### 2.4. Effects of **Oxy** on Resistant ER^+^ Breast Cancer Cells

**Figure 9:**
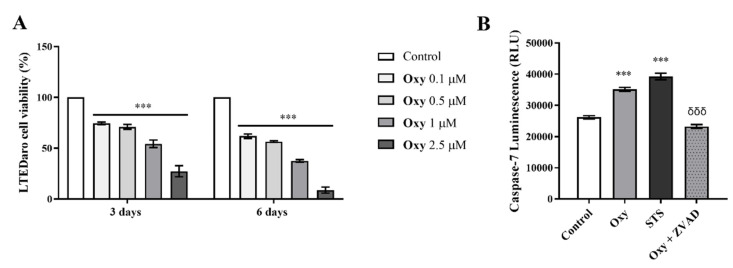
Effects of **Oxy** on viability and cell death of apoptosis of resistant breast cancer cells. (**A**) Effects of **Oxy** (0.1–2.5 μM) on viability of LTEDaro cells after 3 and 6 days. (**B**) Effects of **Oxy** (1 μM) on the activity of caspase-7 after 3 days of treatment. Cells without **Oxy** treatment were considered as control, while cells treated with STS (10 µM) were used as positive control. Z-VAD-FMK was used as a pan-caspase inhibitor. Cell viability effects of **Oxy** were normalized in relation to control (100% of cell viability). Results are the mean ± SEM of at least three independent experiments, each performed in triplicate. For caspase activation assays, results are expressed as relative luminescence units (RLU). Significant differences between **Oxy**-treated cells and the control are shown by *** (*p* < 0.001), while differences between **Oxy**-treated cells with or without Z-VADFMK are indicated by *δδδ* (*p* < 0.001).

In order to deepen the anti-cancer potential of **Oxy**, we explored its effects on viability of resistant breast cancer cells by performing MTT assay, as well as on the involvement of apoptosis by analyzing caspase-7 activity. LTEDaro cells were treated with **Oxy** (0.1–2.5 µM) for 3 and 6 days. As presented in Figure 9, **Oxy** induced a significant (*p* < 0.001) reduction on the viability of LTEDaro cells, with this effect being dose- and time-dependent. A significant (*p* < 0.001) increase in the activity of caspase-7 in the presence of **Oxy** was also noted (Figure 9B). As expected, ZVAD-FMK significantly (*p* < 0.001) reverted the activation of caspase-7 induced by **Oxy**.

## 3. Discussion

Due to the clinical limitations of ER^+^ breast cancer therapy, in the last years many efforts have been made to find new therapeutic strategies or to discover more potent drugs with fewer side effects that may improve treatment. Recently, the interest in finding multi-target compounds for cancer has been rising, as they are more effective, more potent, less toxic and associated with reduced risks of drug interactions [3,25]. In fact, our group has been working on this and we have already discovered potent steroidal AIs that, in addition to aromatase inhibition and/or modulation of aromatase levels, also exhibit ERα- and AR-dependent effects [19] and modulate their expression [21] to induce breast cancer cell death. Moreover, we recently reported a non-steroidal molecule (tamoxifen bisphenol) that also acts as a multi-target compound in ER^+^ breast cancer cells, since it reduces aromatase protein levels and acts as an ERα antagonist and induces ERβ up-regulation to inhibit growth and cause cancer cell death [26]. The ability of an anti-cancer molecule to simultaneously target aromatase, ERs or AR is, from a clinical point of view, very relevant, as these are the main therapeutic targets for these types of tumors. In fact, it should be pointed out that aromatase is the enzyme responsible for estrogen biosynthesis. ERα is responsible for growth, survival and proliferation of breast cancer cells, whereas ERβ display anti-proliferative properties by inhibiting the transcriptional activity of ERα, impairing cell cycle regulation and promoting apoptosis, acting, in that way, as tumor suppressors [3,32,33,34,35]. Depending on the hormonal status, breast cancer setting and cell type, AR may exhibit different roles, such as oncogenic or tumor suppressor [36,37,38,39]. Considering all this, and as an attempt to discover a new steroidal compound with these key properties, the mechanism of action of **Oxy**—a potent steroidal AI, derivative of Exe, designed and synthesized by our group—was investigated [20]. For this AI, it was reported that it affects the viability of ER^+^ breast cancer cells [20] and presents promising anti-cancer properties in lung, liver, colon and prostate cancer cell lines by inducing cell cycle arrest, apoptosis and necrosis as well as DNA damage, and also by inhibiting the DNA damage response [31]. In addition, we previously verified that this molecule is more potent than Exe, the reference steroidal AI used in clinic, with regard to the decrease in cell viability [20,31] and other conventional chemotherapeutic drugs [31]. In this study, our results demonstrate that in sensitive MCF-7aro cells, **Oxy** dramatically affected the rate of DNA synthesis and impaired the progression of cell cycle by causing an arrest on G_0_/G_1_ phase. A similar effect on MCF-7aro cell cycle progression was also reported by our group for Exe [36,40], Ana and Let [41]. However, the G_0_/G_1_ cell cycle arrest caused by **Oxy** was not as pronounced as for the AIs used in the clinic, but was similar to the one induced by the main oxidized Exe metabolite 6-(hydroxymethyl)androsta-1,4,6-triene-3,17-dione (6-HME) [16]. On the other hand, by evaluating different biomarkers of apoptotic cell death, it was observed that **Oxy** induced ∆Ψm loss, translocation of PS to the outer surface of plasma membrane, caused activation of caspases-7, -9 and -8 and increased ROS generation. Thus, **Oxy** promoted apoptosis not only by activating the mitochondrial pathway, but also by activating caspase-8 through an unknown mechanism, with the mitochondria dysfunction being ROS-dependent. A similar behavior has also been reported for Exe in this cell line [36,40], although **Oxy** has an advantage, as in addition to activating the mitochondrial apoptotic pathway, it also activates caspase-8, which may promote a more efficient breast cancer cell death. A cross-talk between the intrinsic pathway and caspase-8 was already detected for other steroidal AIs studied by our group, namely Exe [36,40,42] and its metabolites [16], in sensitive and in resistant ER^+^ breast cancer cells. For the non-steroidal AI Ana, it was also reported that the induction of apoptosis in breast cancer cells occurred via the mitochondrial pathway [41,43,44], but also by the up-regulation of caspase-8 by an unknown mechanism [44]. Several different mechanisms have been described for the interaction between mitochondria and caspase-8 activation [45,46,47,48,49,50,51,52], although this relationship is not totally elucidated. The ability of **Oxy** to impair cell cycle progression and activate apoptosis of cancer cells was also reported by our group, on lung, liver, colon and prostate cancer cell lines [31].

In order to understand the mechanism of action behind these biological effects and also to highlight the multi-target potential of **Oxy**, we investigated the involvement of aromatase, ERα, ERβ and AR. We previously reported that this AI presented an IC_50_ value for anti-aromatase activity of 1.18 µM in MCF-7aro cells and that it affected cell viability in an aromatase-dependent manner, a behavior similar to Exe [20]. Moreover, the IC_50_ values of **Oxy** and Exe on MCF-7aro cells are similar, as the IC_50_ value reported for Exe is 0.9 µM [20]. Nevertheless, contrary to Exe, which acts as an aromatase destabilizer [53], this study demonstrates that its derivative **Oxy** did not affect aromatase expression levels. Despite that, as in an estrogen-enriched environment, **Oxy** continues to affect MCF-7aro cell viability, the involvements of ERα and ERβ on these effects were explored. Our results demonstrated that in the presence of the ERα antagonist ICI or the ERβ antagonist PHTPP, the growth-inhibitory action induced by **Oxy** on MCF-7aro cells was impaired. Thus, these results suggest that **Oxy** acts on cells in an ERα- and ERβ-dependent manner. In fact, it was verified that **Oxy** decreased the gene and protein expression levels of ERα, as well as the levels of ERα-mediated transcription target genes, *TFF1*, *EGR3* and *PDZK1*. In relation to the effects of **Oxy** on Erβ, our data demonstrated that it did not affect the protein levels of ERβ. This comes in line with the absence of ER agonistic activity in the VM7Luc4E2 assay, as well as the apparent antagonism observed under basal conditions, which is most likely a result of the decrease in ERα expression. Importantly, the ER transactivation assays showed that **Oxy** presents a great ER antagonistic potential. Therefore, all these data indicate that this molecule blocks estrogen signaling, acting as a modulator and antagonist of ERα, a mechanism of action typical of SERMs [54]. This molecule does not seem to act as a down-regulator of ERα, since the reduction in ERα protein levels is a consequence of the decrease in the mRNA transcription of *ESR1* gene and not of ERα degradation, as the SERD ICI [55]. Either way, acting as a ERα antagonist is a therapeutic advantage, since by modulating ERα levels and activation, **Oxy** hampers the oncogenic properties of this receptor, impairing growth and proliferation of breast cancer cells [3,34,35]. This behavior induced by **Oxy** is an advantage in relation to Exe, since it is known that Exe presents weak estrogen-like effects in breast cancer cells [41,56]. In fact, we recently reported that besides reducing the expression levels of ERα on MCF-7aro cells, Exe does not affect the transcription levels of *ESR1* and *EGR3* genes, with this latter effect related to the weak-estrogen like effect induced by Exe [41]. Moreover, the ability of **Oxy** to decrease *EGR3* transcription levels is a clinical benefit, as this gene is correlated with poor response to therapy and decreased disease-free survival and overall survival [57]. In addition, the effects induced by **Oxy** on ERα are also an advantage over Ana and Let, since these non-steroidal AIs increase the expression of ERα at gene and protein levels on breast cancer cells [41]. Recently, clinical data proposed a relationship between leptin levels and the hormonal effects induced by the AIs Exe and Let [58]. Moreover, in MCF-7 cells, it was suggested that leptin may induce a functional activation of ERα through the ERK_1/2_ pathway [59]. As Exe and **Oxy** present a similar steroidal chemical structure, **Oxy** may modulate leptin signaling, as reported for Exe [59], and thus may indirectly affect ERα expression and activation. In addition, considering the lack of cell death in the co-exposure with the ERβ antagonist PHTPP, our results also suggest that **Oxy** can act through ERβ to induce breast cancer cell death. Acting as an agonist of ERβ, it is also a therapeutic advantage, since ERβ is considered a breast tumor suppressor [3,32,33,35]. Nevertheless, it should be pointed that PHTPP exhibits 36-fold more selectivity for ERβ than ERα and that it abrogates estrogen action by acting on ERβ, presenting minimal effects through ERα [60].

It is known that AR is expressed in 85–95% of ER^+^ breast cancer cases and in 77% of invasive breast cancers [61]. Furthermore, AR may have different functions after AI therapy. In fact, for the non-steroidal AI letrozole [41,62] and C7- [19] and C6-substituted steroidal AIs, [21] AR presents a pro-death role, while for the steroidal AI Exe it exhibits pro-survival and oncogenic functions [36]. Thus, considering the importance of AR in this type of breast tumor and the dual role of AR function after AI therapy, it is important to understand the involvement and the role of AR on **Oxy** action. Our results indicate that **Oxy** acts on cells in an AR-dependent manner, since the **Oxy** growth–inhibitory effect is compromised when AR is blocked by CDX, being observed an inhibition of apoptosis. Moreover, **Oxy** has the ability to increase the expression levels of AR and, using the AR-EcoScreen™ assay, we further showed that **Oxy** detains agonistic activity towards AR. Therefore, all these results indicate that **Oxy** modulates AR, acting as an AR agonist, which leads to ER^+^ breast cancer cell death. This is a therapeutic advantage, as AR acts as a breast tumor suppressor, a mechanism of action similar to the observed for the non-steroidal AI letrozole [41,62] and to other C7- [19] and C6-substituted steroidal AIs [21] synthesized by our group. Interestingly, these AR-associated pro-death effects are not observed with Exe, as in this case, the AR has an oncogenic role [36].

Additionally, **Oxy** was able to re-sensitize the resistant ER^+^ breast cancer cells, LTEDaro cells, since it was able to strongly reduce cell viability and increase the activity of the effector caspase-7. This behavior is an advantage of **Oxy** in relation to the AIs used in the clinic, since this cell model is characterized by mimicking long-term resistance to the AIs used in the clinic [16,17,36,42,63,64,65]. On the other hand, the effects induced by **Oxy** on this resistant cell line are much more pronounced and appealing than those induced by the Exe metabolites 6-HME and 17β-hydroxy-6-methylenandrosta-1,4-dien-3-one (17-βHE) [16].

In conclusion, to the best of our knowledge, this is the first work that describes an anti-cancer molecule, **Oxy**, that in addition to acting as an AI, also modulates both ERs and AR acting as an ERα antagonist and AR agonist, rendering **Oxy** a more effective anti-tumor profile. In fact, from a clinical point of view, these multi-target properties are very relevant for this type of tumor since they correspond to therapeutic targets with key roles in cancer growth, survival or promotion of cancer cell death. Thus, by acting as an AI that also modulates ERα, ERβ and AR, **Oxy** impaired cancer cell growth by disrupting cell cycle and DNA synthesis, and induced apoptosis of sensitive ER^+^ breast cancer cells. This multi-target action of **Oxy** on ERs and AR is an advantage over the three AIs used in clinic, since none of these AIs act on these receptors in a therapeutically beneficial way. Additionally, it should be pointed that all these anti-cancer properties were observed at a dose ten times lower than the doses used in similar pre-clinical studies for the three AIs used in clinic [16,36,40,41]. Moreover, we had previously demonstrated that **Oxy** is more potent than Exe with regard to the decrease in cell viability [20,31] and even more potent than other conventional chemotherapeutic drugs [31]. In addition, this multi-target compound also has the ability to re-sensitize resistant breast cancer cells by activating apoptosis. All these findings emphasize the therapeutic potential of **Oxy**. Thus, this work, besides highlighting the importance of multi-target compounds for ER^+^ breast cancer subtype, also allowed the discovery of a promising multi-target anti-cancer molecule for ER^+^ breast cancer treatment, **Oxy**.

## 4. Material and Methods

### 4.1. Compound under Study

In this work, we studied the steroidal aromatase inhibitor (AI) 1α,2α-epoxy-6-methylenandrost-4-ene-3,17-dione, which was previously synthesized by our group [20] and further designated as Oxymestane-D1 (**Oxy**).

### 4.2. Cell Culture

In this study, two different ER^+^ breast cancer cell lines were used to investigate the in vitro effects of AI **Oxy**, the sensitive MCF-7aro cells and the resistant LTEDaro cells. MCF-7aro cells are an ER^+^ aromatase-overexpressing human breast cancer cell line, obtained from the parental human epithelial ER^+^ breast cancer cell line (MCF-7 cells), after stable transfection with the human placental aromatase gene using Geneticin (G418) selection, as previously described [66,67]. These cells mimic the tumor microenvironment, being thus considered a suitable in vitro cell model to study ER^+^ breast cancer and AIs [44]. Cells were maintained with Eagle’s minimum essential medium (MEM) (Gibco Invitrogen Co., Paisley, Scotland, UK) supplemented with 1 mmol/L sodium pyruvate, 1% penicillin-streptomycin-amphotericin B, 100 μg/mL G418 and 10% heat-inactivated fetal bovine serum (FBS) (Gibco Invitrogen Co., Paisley, Scotland, UK). Three days before the experiments, cells were cultured in an estrogen-free MEM without phenol-red (Gibco Invitrogen Co., Paisley, Scotland, UK), containing 5% pre-treated charcoal heat-inactivated fetal bovine serum (CFBS), 1 mmol/L of sodium pyruvate, 1% of penicillin-streptomycin-amphotericin B and 2 mmol/L of l-glutamine (Gibco Invitrogen Co., Paisley, Scotland, UK), to avoid the interference of the hormones present in FBS and of the estrogen-like effects of phenol-red, as previously reported [36,40]. After this period, cells were stimulated with testosterone (T) or with estradiol (E_2_) (Sigma-Aldrich Co., Saint Louis, MI, USA), the aromatase substrate or the aromatase product, respectively, at 1 nM, which were used as proliferation inducing agents [40,68] and treated with **Oxy**. All the experiments were performed under these conditions.

The long-term estrogen-deprived human ER^+^ breast cancer cell line, LTEDaro cells, mimics the late-stage of acquired resistance to the AIs used in clinic, since they originated through long-term estrogen deprivation of the parental MCF-7aro cells, being thus considered a suitable in vitro cell model to study resistance [36,63,64]. These cells were cultured in Eagle’s MEM without phenol-red and supplemented with Earle’s salts and with 1 mmol/L sodium pyruvate, 1% penicillin–streptomycin-amphotericin B, 1% l-Glutamine, 100 μg/mL G418, and 10% of CFBS, as previously reported [36,65]. For the assays, cells were cultured in these conditions and treated with **Oxy**.

Both cell lines were kindly provided by Professor Shiuan Chen (Beckman Research Institute, City of Hope, Duarte, CA, USA) and were maintained at 37 °C and 5% CO_2_ atmosphere.

Stock solutions of T and E_2_ were prepared in absolute ethanol (Sigma-Aldrich Co., Saint Louis, MI, USA). On the other hand, **Oxy**, Exemestane (Exe) (Sigma-Aldrich Co., Saint Louis, MI, USA), ICI 182,780 (Sigma-Aldrich Co., Saint Louis, MI, USA), Casodex (CDX) (Sigma-Aldrich Co., Saint Louis, MI, USA) and 4-[2-phenyl-5,7,bis(trifluoromethyl)pyrazol [1,5-a]pyrimidin-3-yl]phenol (PHTPP) (Sigma-Aldrich Co., Saint Louis, MI, USA) were prepared in 100% DMSO (Sigma-Aldrich Co., Saint Louis, MI, USA). The AI **Oxy**, as well as, T, E_2_, Exe, ICI, PHTPP and CDX were stored at −20 °C, and fresh dilutions were prepared in medium before each experiment. Final concentrations of ethanol and DMSO in culture medium were lower than 0.05% and 0.01%, respectively. All the controls contained these vehicles in these culture conditions.

### 4.3. Cell Viability and Cell Proliferation

The sensitive MCF-7aro and the resistant LTEDaro cells were cultured in 96-well plates, with a cellular density of 2.5 × 10^4^ cells/mL (2 and 3 days) and 1 × 10^4^ cells/mL (6 days), and incubated with different concentrations of **Oxy** (0.1–5 µM) during 2, 3 and 6 days. Depending on the type of analysis, MCF-7aro cells were incubated with T (1 nM) or E_2_ (1 nM), as well as, with ICI 182,780 (100 nM), PHTPP (1 µM) or CDX (1 µM). Cells without **Oxy** treatment were considered as control.

To explore the effects of **Oxy** on the viability of LTEDaro cells, the tetrazolium salt, 3-(4,5-dimethylthiazol-2-yl)-2,5-difenyltetrazolium (MTT) assay was performed. After each incubation time, MTT (0.5 mg/mL) (Sigma-Aldrich Co., Saint Louis, MI, USA) was added and quantified spectrophotometrically in a Biotek Synergy HTX Multi-Mode Microplate Reader (Biotek Instruments, Winowski, VT, USA).

To study the effects of **Oxy** on DNA synthesis of MCF-7aro cells, the ^3^H-thymidine incorporation assay was performed. At each exposure time and for the final 8 h, ^3^H-thymidine (0.5 µCi) (Amersham International, Amersham, UK) was added to each well. Cells were further harvested using a semi-automated cell harvester (Skatron Instruments, Lier, Norway), scintillation cocktail was added, and ^3^H-thymidine incorporation was determined in a scintillation counter (LS 6500, Beckman Instruments, Brea, CA, USA).

All the results are expressed as relative percentage of the untreated control cells (100% of cell viability and cell proliferation).

### 4.4. Cell Cycle Analysis

To investigate the effects of **Oxy** on MCF-7aro cell cycle progression, the DNA content was assessed by flow cytometry. MCF-7aro cells (7 × 10^5^ cells/mL) stimulated with T (1 nM) were incubated with **Oxy** (1 and 2.5 µM) for 3 days. Cells only treated with T were considered as control. After the incubation period, cells were fixed with 70% cold ethanol and stained with a DNA staining solution (5 µg/mL Propidium Iodide (PI), 0.1% Triton X-100 and 200 µg/mL DNase-free RNase A (Sigma-Aldrich Co., Saint Louis, MI, USA)), as previously described [19]. DNA content was analyzed by flow cytometry based on the acquisition of 40 000 events in a BD Accuri™ C6 cytometer (San Jose, CA, USA), equipped with a BD Accuri™ C6 analysis software. Detectors for the three fluorescence channels (FL-1, FL-2 and FL-3) and for forward (FSC) and side (SSC) light scatter were set on a linear scale. Debris, cell doublets and aggregates were gated out using a two-parameter plot of FL-2-Area to FL-2-Width of PI fluorescence. Data were analyzed using the BD Accuri™ C6 analysis software. The anti-proliferative effects were indicated by the percentage of cells in G_0_/G_1_, S and G_2_/M phases of the cell cycle.

### 4.5. Analysis of Apoptosis

To understand the involvement of apoptosis on sensitive and resistant treated cells, the mitochondrial transmembrane potential (ΔΨm), the activation of caspases-9, -8 and -7, the production of intracellular reactive oxygen species (ROS), as well as the translocation of phosphatidylserine (PS) were studied.

MCF-7aro and LTEDaro cells (7 × 10^5^ cells/mL) were cultured in 6-well plates and treated with **Oxy** (1 μM) with or without CDX (1 µM) for 3 days. MCF-7aro cells were stimulated with T (1 nM). Cells without **Oxy** treatment were designated as control, while cells treated with Staurosporine (STS) (10 μM), carbonyl cyanide m-chlorophenylhydrazone (10 μM), or with phorbol 12-myristate 13-acetate (PMA) (25 ng/mL) (Sigma-Aldrich Co., Saint Louis, MI, USA) were used as positive controls.

The mitochondrial transmembrane potential (ΔΨm) was evaluated by flow cytometry using 3,3′-dihexyloxacarbocyanine iodide (DiOC_6_(3)) (Gibco Invitrogen Co., Paisley, Scotland, UK) at 10 nM, as previously described [19]. PI at 5 μg/mL was added prior to flow cytometry to discriminate between live cells that stain only with DiOC_6_(3) (DiOC_6_(3)^+^/PI^−^), early apoptotic cells that lost the ability to accumulate DiOC6(3) (DiOC_6_(3)^−^/PI^−^), and late apoptotic/necrotic cells that stained only with PI (DiOC_6_(3)^−^/PI^+^). Flow cytometric analysis based on the acquisition of 40,000 events was carried out in a BD Accuri™ C6 cytometer (San Jose, CA, USA), equipped with a BD Accuri™ C6 analysis software. Detectors for FSC and SSC light scatter were set on a linear scale and all three fluorescence channels (FL-1, FL-2 and FL-3) were set on a logarithmic scale. FL-1 was used to measure DiOC_6_(3) at green fluorescence, while FL-2 and FL-3 were used to measure PI red fluorescence. Data were analyzed using BD Accuri™ C6 analysis software.

To study translocation of PS, cells were labelled with Annexin V-FITC Apoptosis Detection Kit (BioLegend Way, San Diego, CA, USA), according to the manufacturer’s instructions, and analyzed based on the acquisition of 40,000 events in the BD Accuri™ C6 cytometer (San Jose, CA, USA), equipped with BD Accuri™ C6 analysis software, as previously described [36]. Detectors for all three fluorescence channels (FL-1, FL-2 and FL-3) were set on a logarithmic scale. Bivariant analysis of Annexin-FITC fluorescence (FL-1) and 7-amino-acitomycin (7-AAD) fluorescence (FL-3) distinguished different cell populations: Annexin V^−^/7-AAD^−^ were considered as viable cells, Annexin V^+^/7-AAD^−^ corresponded to apoptotic cells and Annexin V^+^/7-AAD^+^ were designated as late apoptotic and necrotic cells. Data were analyzed using BD Accuri™ C6 analysis software.

To evaluate caspase activities, luminescent assays with Caspase-Glo^®^ 9, Caspase-Glo^®^ 8 and Caspase-Glo^®^ 3/7 (Promega Corporation, Madison, WI, USA), were performed according to the manufacturer’s instructions and as previously described [18]. As a negative control, the pan-caspase inhibitor Z-VAD-FMK (50 μM) (Sigma-Aldrich Co., Saint Louis, MI, USA) was used. The resultant luminescence was measured in a Biotek Synergy HTX Multi-Mode Microplate Reader (Biotek Instruments, Winowski, VT, USA) and results were presented as relative luminescence units (RLU).

To detect the levels of intracellular ROS, the 2′,7′-dichlorodihydrofluorescein diacetate (DCFH_2_-DA) method was used by labelling cells with DCFH_2_-DA (50 μM) (Sigma-Aldrich Co., Saint Louis, MI, USA), as previously described [18]. Fluorescence was measured using an excitation wavelength of 480 nm and an emission filter of 530 nm in the Biotek Synergy HTX Multi-Mode Microplate Reader (Biotek Instruments, Winowski, VT, USA) and data were presented as mean fluorescence intensity (MFI).

### 4.6. Western Blot Analysis

The expression levels of aromatase, estrogen receptor α (ERα), estrogen receptor β (ERβ) and androgen receptor (AR) were evaluated by Western Blot. For this purpose, MCF-7aro cells (7 × 10^5^ cells/mL) were cultured in 6-well plates and incubated with **Oxy** (1 μM), during 8 h to study aromatase expression [19,21], and for 3 days to study the expression of ERα, ERβ and AR [26,36]. Cells without **Oxy** treatment were designated as control, while cells treated with Exe (10 µM), ICI 182 780 (100 nM), and PHTPP (1 µM) were used as positive controls. After treatment, cells were collected as previously reported [40] and 50 μg of protein sample was subjected to 10% of SDS-PAGE and then transferred onto nitrocellulose membranes. For the immunodetection, the mouse monoclonal CYP19A1 (1:200, sc-374176), mouse monoclonal ERα (1:200, sc-8002), mouse monoclonal AR (1:200, sc-7305) (Santa Cruz Biotechnology, Santa Cruz, CA, USA) and mouse monoclonal ERβ (1:200, PPZ0506) (Thermo Fisher, Waltham, MA, USA) were used as primary antibodies, whereas the peroxidase-conjugated goat anti-mouse (1:2000, G21040) (Thermo Fisher, Waltham, MA, USA) was used as a secondary antibody. A mouse monoclonal anti-β-tubulin antibody (1:500, sc-5274) (Santa Cruz Biotechnology, Santa Cruz, CA, USA) was used to control loading variations. Immunoreactive bands were visualized using a chemiluminescent substrate Super Signal West Pico (Pierce, Rockford, IL, USA) in a ChemiDoc™ Touch Imaging System (Bio-Rad, Laboratories Melville, NY, USA).

### 4.7. RNA Extraction and qPCR

Quantitative polymerase chain reaction (qPCR) analysis was performed to investigate the effects of **Oxy** on MCF-7aro cells in the transcription levels of *ESR1*, *EGR3*, *PDZK1* and *TFF1* genes, as previously reported [41]. MCF-7aro cells (7×10^5^ cells/mL) were cultured in 6-well plates, stimulated with T (1 nM) and incubated with **Oxy** (1 μM) for 3 days. Cells without **Oxy** treatment were designated as control, while cells treated with ICI 182 780 (100 nM) were used as positive control. The RNA was extracted using the TripleXtractor reagent (GRiSP Research Solutions, Porto, Portugal), according to the manufacturer’s protocol. RNA quality was measured with the Experion RNA StdSens Kit (Bio-Rad Laboratories), in the Experion analytical software (Bio-Rad Laboratories), and quantified using NanoDrop ND-1000 Spectrophotometer (NanoDrop Technologies, Inc, Wilmington, DE, USA). RNA was further converted into cDNA using the GRiSP Xpert cDNA Synthesis Mastermix (GRiSP Research Solutions, Porto, Portugal), containing reverse transcriptase, according to manufacturer’s protocol. cDNA was amplified using GRiSP Xpert Fast SYBR (GRiSP Research Solutions, Porto, Portugal) in MiniOpticon Real-Time PCR Detection System (Bio-Rad Laboratories, Hercules, CA, USA), according to the manufacturer’s protocol. Primer sequences (5′-3′) are presented in Table 4. The fold change in gene expression was calculated using the 2^−ΔΔCt^ method, using as housekeeping genes, *TUBA1A* and *ACTB*.

### 4.8. AR and ER Transactivation Assays

In order to assess potential ER activity of **Oxy**, the VM7Luc ER transactivation assay, from Test No. 455 of the OECD Guidelines for the Testing of Chemicals [69], was performed. This assay uses VM7Luc4E2 cells, derived from the MCF-7 cell line, which endogenously express both human ERα and ERβ forms. Cells were maintained in Roswell Park Memorial Institute 1640 medium, supplemented with 1% penicillin/streptomycin, and 8% heat-inactivated FBS. Three days before the experiments, cells were split into an estrogen-free Dulbecco’s Modified Eagle Medium (DMEM) without phenol red, supplemented with 1% penicillin/streptomycin, 4.5% CFBS, 2% L-glutamine, and 110 mg/mL sodium pyruvate (Gibco Invitrogen Co., Paisley, Scotland, UK). This medium was further used for seeding and exposure of cells. Briefly, cells (4 × 10^5^ cells/mL) were exposed to 0.1–1 µM **Oxy** in 96-well white plates for 24 h. This exposure was conducted in the absence of any ER agonist to assess potential ER agonistic activity of **Oxy**, or in the presence of 1 nM T to assess antagonistic activity. Luminescence was read in EnSpire^®^ multimode plate reader (Perkin Elmer, Inc., Waltham, MA, USA) using the Steady-Glo^®^ Luciferase Assay System (Promega Corporation, Madison, WI, USA). ATP levels were assessed as an indirect measure of cell viability, using the CellTiter-Glo^®^ Luminescent Cell Viability Assay (Promega Corporation, Madison, WI, USA). T-treated cells (781.2 pM–25.6 µM) were used as a positive control of ER agonism, with a maximum effect of 12.7-fold increase over control. Raloxifene (12.0 pM–24.5 nM; Biosynth Ltd., Berkshire, United Kingdom) was used as a positive control of ER antagonism, with a maximum inhibitory effect of 42.4% in the presence of 1 nM T.

To confirm the potential activity of **Oxy** towards AR, the AR-EcoScreen™ assay, from Test No. 458 of the OECD Guidelines for the Testing of Chemicals [70], was conducted. The AR-EcoScreen™ cell line is derived from the Chinese hamster ovary CHO-K1 cell line, and it expresses three stably inserted constructs: the human AR; a firefly luciferase reporter construct bearing an androgen responsive element gene; and a renilla luciferase reporter construct for simultaneous assessment of cell viability. Cells were maintained in DMEM/Nutrient Mixture F-12 (DMEM/F12) medium without phenol red, supplemented with 1% penicillin/streptomycin, 10% heat-inactivated FBS, 200 µg/mL zeocin, and 100 µg/mL hygromycin. Cells were seeded (9 × 10^4^ cells/mL) onto 96-well white plates and exposed to 0.1–1 µM **Oxy** for 24 h. Seeding and exposure of cells was performed in DMEM/F12 without phenol red, containing 1% penicillin/streptomycin and 5% CFBS. The exposure was conducted in the absence or presence of the potent AR agonist 0.1 nM methyltrienolone (R1881; AbMole BioScience, Houston, TX, USA) to assess potential AR agonistic and antagonistic activity of **Oxy**, respectively. Luminescence for both AR activity and cell viability was read in EnSpire^®^ multimode plate reader (Perkin Elmer, Inc., Waltham, MA, USA) using the Dual-Glo^®^ Luciferase Assay System (Promega Corporation, Madison, WI, USA). R1881 (7.8 pM–1 nM) was used as positive a control of AR agonism, showing a maximum effect of 5.5-fold increase over control. Hydroxyflutamide (OHF; 4.1 nM–9 µM; Sigma-Aldrich Co., Saint Louis, MI, USA) was used as positive control of AR antagonism, with a maximum inhibition of 69.8% in the presence of 0.1 nM R1881.

Data from four independent experiments were presented as fold change compared to control, which was set as 1. Stock solutions of T, raloxifene, R1881 and OHF were prepared in 100% DMSO and stored at −20 °C. Dilutions were prepared freshly in medium before each experiment. The final concentration of DMSO in exposure medium for all conditions was 0.06%.

### 4.9. Statistical Analysis

All the assays were performed in triplicate in at least three independent experiments, and data were expressed as the mean ± SEM. Statistical analysis was performed by using GraphPad Prism 7^®^ software (GraphPad Software, Inc., San Diego, CA, USA) and by applying the analysis of variance (ANOVA), followed by multiple comparisons using Tukey and Bonferroni post hoc tests. Values that were *p* < 0.05 were designated as statistically significant.

## Figures and Tables

**Table 1:** Effects of **Oxy** on MCF-7aro cell cycle progression.

	G_0_/G_1_	S	G_2_/M
Testosterone	74.29 ± 0.68	11.34 ± 0.77	12.43 ± 0.74
T + **Oxy** (1 µM)	82.32 ± 0.72 ***	5.20 ± 0.20 ***	9.60 ± 0.89
T + **Oxy** (2.5 µM)	83.78 ± 0.58 ***	4.52 ± 0.37 ***	8.90 ± 0.65 *

Cells stimulated with T (1 nM) were treated with **Oxy** at 1 and 2.5 μM for 3 days. Cells were stained with PI (1 μg/mL) and analyzed by flow cytometry. Values are expressed as a percentage of single cell events in each stage of the cell cycle and are the mean ± SEM of at least three independent experiments, all performed in triplicate. Significant differences between the control and treated cells are shown by * (*p* < 0.01) and *** (*p* < 0.001).

**Table 2:** Effects of **Oxy** on mitochondrial transmembrane potential (Δψm) in MCF-7aro cells.

	Viable Cells	Cells with Δψm Loss
Testosterone	94.66 ± 0.79	5.35 ± 0.78
T + **Oxy** (1 µM)	69.74 ± 0.74 ***	30.27 ± 0.73 ***
T + CCCP (10 µM)	41.43 ± 1.24 ***	58.56 ± 1.23 ***

Cells were stimulated with T (1 nM) and incubated with **Oxy** (1 µM) for 3 days. Treated cells were harvested and labelled with DiOC_6_(3) and PI followed by flow cytometry analysis. Data are presented as % of viable cells and cells with Δψm loss. Cells only cultured with T were considered as control and cells treated with T plus CCCP (10 µM) were considered as positive control. The data represent means ± SEM of three independent experiments conducted in triplicate. The ratio treatment/control is presented in bold within brackets. Significant differences between the control versus treated cells are indicated by *** (*p* < 0.001).

**Table 3:** Effects of **Oxy** on Annexin V-FITC labelling in MCF-7aro cells.

	AnnexinV^−^/7-AAD^−^	AnnexinV^+^/7-AAD^−^	AnnexinV^+^/7-AAD^+^
Testosterone	85.45 ± 0.22	4.96 ± 0.73	8.70 ± 0.78
T + **Oxy** (1 µM)	61.74 ± 1.15 ***	15.93 ± 1.02 (3.21) ***	24.13 ± 0.87 ***
T + STS (10 µM)	59.20 ± 5.50 ***	21.99 ± 0.76 (4.43) ***	19.84 ± 2.76 *

Cells stimulated with T (1 nM) and incubated with **Oxy** (1 µM) for 3 days were labeled with Annexin V-FITC and 7-AAD followed by flow cytometry analysis. Data are presented as % of viable cells (Annexin V^−^/7-AAD^−^), % of early apoptotic (Annexin V^+^/7-AAD^−^) and late apoptotic or necrotic cells (Annexin V^+^/7-AAD^+^). Cells only treated with T were considered as control, while cells treated with STS (10 µM) were considered as positive control for apoptosis. The results are expressed as mean ± SEM of three independent experiments, performed in triplicate. The ratio treatment/control is presented in bold within brackets. Significant differences among the control and treated cells are denoted by * (*p* < 0.05), *** (*p* < 0.001).

**Table 4:** Primer sequences and qPCR conditions for housekeeping and target genes.

Target Gene	Primer Sequences (5′-3′)		Ta/°C
	Sense	Anti-Sense	
*ESR1*	CCTGATCATGGAGGGTCAAA	TGGGCTTACTGACCAACCTG	55
*EGR3*	GACTCCCCTTCCAACTGGTG	GGATACATGGCCTCCACGTC	56
*TFF1*	GTGGTTTTCCTGGTGTCACG	AGGATAGAAGCACCAGGGGA	55
*PDZK1*	ACTCTGCAGGCTGGCTAAAG	ACCGCCCTTCTGTACCTCTT	56
*ACTB*	TACAGCTTCACCACCACAGC	AAGGAAGGCTGGAAGAGAGC	55
*TUBA1A*	CTGGAGCACTCTGATTGT	ATAAGGCGGTTAAGGTTAGT	55

## Data Availability

Not applicable.

## Sample Availability

Samples of the compound Oxymestane-D1 (**Oxy**) are available from the authors.
